# Implementation of an interactive training module on hand hygiene

**DOI:** 10.1186/2047-2994-4-S1-P289

**Published:** 2015-06-16

**Authors:** L Qalla-Widmer, M Attinger, R Blanc, I Amherdt, F Boiron, D Zbinden, C Petignat

**Affiliations:** 1 Unité HPCI, CHUV, Lausanne, Switzerland; 2 PCI, Hôpital Riviera Chablais, Vevey, Switzerland; 3 Collectif Hygiène des mains, Fédération des Hôpitaux vaudois, Prilly, Switzerland; 4 SMPH, Lausanne, Switzerland; 5 SMPH Unité HPCI, CHUV, Lausanne, Switzerland

## Introduction

In most cases, hands of caregivers are the vehicle for the transmission of germs to the patient. Therefore, it is crucial to help medical staff and healthcare workers acquiring the skills necessary for optimal hand hygiene (HH) in order to ensure quality of care and patient safety.

## Objectives

To give the caregivers a training tool well adapted to their needs and integrated into a blended learning (class and distance).

## Methods

The Hygiene Prevention and Infection Control (HPCI) Unit - Vaud, the Preventive Medicine Service of Center Hospital Universitary of Vaud (SMPH) in partnership with the collective of the Federation of Vaud Hospital, the Commission e-learning of University to Lausanne, the Center for Education and Audiovisual Communication, have developed an interactive training module of HH in 3 steps.

### Conception

- Identification of the academic content

- Development of a teaching strategy fitted to any learning styles

- Selection of the interactive teaching resources

### Development

- Development of academic learning content

- Development of practical content (care situations cut out in various interactive e-lessons)

- Tutorial construction (integration of multimedia elements and interactivity)

### Implementation

- Module overview by infection control professionals in Vaud institutions

- Module provision through training platforms (Moodle, MyTeacher)

- The Module is freely accessible on the Internet

## Results

After a brief theoretical reminder (common to all healthcare workers), the module content covers aspects of skills through simulation exercises. Care situations are specific for each category of professionals (doctors, nurses). Throughout the course, a knowledge assessment evaluates the benefits of training.

## Conclusion

The training module meets the predefined goals. This e-learning module broadens infection control class offer and is part of the five areas of the WHO multimodal HH strategy. The educational effectiveness will be evaluated after 9-12 months by a query filled up by the caregivers, by the participation rate, and by reality in practice (assessed during audits).

## Disclosure of interest

None declared.

